# Editorial: New insights of immune cells in cardiovascular and metabolic disorders

**DOI:** 10.3389/fimmu.2023.1282078

**Published:** 2023-09-28

**Authors:** Yingmei Feng, Xiangwei Xiao, Catherine Verfaillie

**Affiliations:** ^1^ Beijing Youan Hospital, Capital Medical University, Beijing, China; ^2^ Children’s Hospital of Pittsburgh, School of Medicine, University of Pittsburgh, Pittsburgh, PA, United States; ^3^ KU Leuven, Leuven, Belgium

**Keywords:** cardiovascular, metabolic, immune cells, insight, diabetes

The prevalence of metabolic disorders has increased gradually during recent decades. Such disorders are potent risk factors for cardiovascular disease, diabetes, and non-alcoholic fatty liver disease. Therefore, how to control cardiovascular and metabolic disorders has become a global target for disease prevention and treatment.

Despite the distinct features of each disease, there is compelling evidence for the critical roles of immune cells in the pathogenesis of cardiovascular and metabolic disorders (1–3). For instance, CD4^+^ T cells are present in atherosclerotic plaques (1); nevertheless, these cells exist in a variety of subtypes, and the involvement of each in plaque progression has yet to be characterized. Nonetheless, the CANTOS trial of 10,061 patients who had previously experienced myocardial infarction demonstrated that blockade of the NLRP3–IL-1–IL-6 signaling pathway using the IL-1β neutralizing antibody canakinumab significantly reduced the incidence of adverse cardiovascular events when compared with the placebo group (4). An ongoing randomized double-blind placebo-controlled phase I/II clinical trial (LILACS) is evaluating the therapeutic efficacy of low-dose IL-2 for the treatment of patients with stable ischemic heart disease and acute coronary syndromes (5). In addition, the efficacy of Treg adoptive transfers followed by low-dose IL-2 administration is being tested in patients with type 1 diabetes (6).

To gain insight into how the immune system participates in cardiovascular and metabolic disorders, the present Research Topic, entitled “*New insights into the roles of immune cells in cardiovascular and metabolic disorders*,” represents a collection of original research studies and reviews. Through the immunostaining of autopsy samples of adult hearts with non-ischemic cardiomyopathy, ischemic cardiomyopathy, and controls, Bermea et al. identified abundant naive B cells in the interstitium of human hearts. By analyzing publicly available spatial transcriptomic and single-cell sequencing datasets of myocardial and peripheral blood mononuclear cells, they were also able to show that the function of myocardial B cells is conserved across species. Taking advantage of an IL-10-deficient human-like hamster model, Shi et al. showed that IL-10 deficiency disrupts triglyceride and cholesterol distribution in lipoprotein particles and therefore impairs cholesterol transport and accelerates the progression of atherosclerosis. In addition, by employing laser capture microdissection, they identified epigenetic regulators of endothelial cells and macrophages in patients with diabetes and atherosclerotic plaques. Finally, Huang et al. revealed that histone deacetylase 3 modulates the inflammatory and metabolic properties of macrophages and accelerates the progression of atherosclerosis in diabetes.

Taken together, these articles add to knowledge of the roles of immune cells in cardiovascular and metabolic disorders. Moreover, they highlight avenues for future research and suggest potential future treatment strategies.

## Author contributions

YF: Writing – original draft. XX: Writing – review & editing. CV: Writing – review & editing.
